# Author Correction: Strategical selection of maintenance type under different conditions

**DOI:** 10.1038/s41598-023-44336-8

**Published:** 2023-10-17

**Authors:** Mohammad M. Hamasha, Ala H. Bani‑Irshid, Sahar Al Mashaqbeh, Ghada Shwaheen, Laith Al Qadri, Mohammad Shbool, Dania Muathen, Mussab Ababneh, Shahed Harfoush, Qais Albedoor, Adnan Al‑Bashir

**Affiliations:** 1 Department of Industrial Engineering, Faculty of Engineering, The Hashemite University, Zarqa, 13133 Jordan; 2 Department of Industrial Engineering, Faculty of Engineering, The University of Jordan, Amman, 11942 Jordan

Correction to: *Scientific Reports* 10.1038/s41598-023-42751-5, published online 20 September 2023

The original version of this Article contained an error in Affiliation 2, which was incorrectly given as ‘Department of Industrial Engineering, Faculty of Engineering, Jordan University, Amman 11942, Jordan’. The correct affiliation is listed below:

Department of Industrial Engineering, Faculty of Engineering, The University of Jordan, Amman 11942, Jordan.

The original Article has been corrected.

